# Anticancer Activities of Meroterpenoids Isolated from the Brown Alga *Cystoseira usneoides* against the Human Colon Cancer Cells HT-29

**DOI:** 10.3390/foods9030300

**Published:** 2020-03-06

**Authors:** Hanaa Zbakh, Eva Zubía, Carolina De Los Reyes, José M. Calderón-Montaño, Virginia Motilva

**Affiliations:** 1 Department of Pharmacology, Faculty of Pharmacy, University of Seville, 41012 Seville, Spain; ha.zbakh@gmail.com (H.Z.); jcalderon@us.es (J.M.C.-M.); 2 Department of Biology, Faculty of Sciences, University of Abdelmalek Essaâdi, Tetouan 93000, Morocco; 3 Department of Organic Chemistry, Faculty of Marine and Environmental Sciences, University of Cadiz, 11510 Puerto Real (Cádiz), Spain; eva.zubia@uca.es (E.Z.); carolina.dereyes@uca.es (C.D.L.R.)

**Keywords:** meroterpenoids, *Cystoseira usneoides*, colon cancer, HT-29, cell cycle, apoptosis, metastasis

## Abstract

Colorectal cancer (CRC) is one of the most common types of cancers and a leading cause of cancer death worldwide. The current treatment for CRC mainly involves surgery, radiotherapy, and chemotherapy. However, due to the side effects and the emergence of drug resistance, the search for new anticancer agents, pharmacologically safe and effective, is needed. In the present study, we have investigated the anticancer effects of eight algal meroterpenoids (AMTs, **1-8**) isolated from the brown seaweed *Cystoseira usneoides* and their underlying mechanisms of action using HT-29, a highly metastatic human colon cancer cell line. All the tested meroterpenoids inhibited the growth of HT-29 malignant cells and were less toxic towards non-cancer colon cells, with the AMTs **1** and **5** exhibiting selectivity indexes of 5.26 and 5.23, respectively. Treatment of HT-29 cells with the AMTs **1**, **2**, **3**, **4**, **5**, and **7** induced cell cycle arrest in G2/M phase and, in some instances, apoptosis (compounds **2**, **3**, and **5**). Compounds **1**-**8** also exhibited significant inhibitory effects on the migration and/or invasion of colon cancer cells. Mechanistic analysis demonstrated that the AMTs **1**, **2**, **5**, **6**, **7**, and **8** reduced phosphorylation levels of extracellular signal-regulated kinase (ERK) and the AMTs **2**, **3**, **4**, **5**, **7**, and **8** decreased phosphorylation of c-JUN N-terminal kinase (JNK). Moreover, the AMTs **1**, **2**, **3**, **4**, **7**, and **8** inhibited phosphorylation levels of protein kinase B (AKT) in colon carcinoma cells. These results provide new insights into the mechanisms and functions of the meroterpenoids of *C. usneoides*, which exhibit an anticancer effect on HT-29 colon cancer cells by inducing cell cycle arrest and apoptosis via the downregulation of ERK/JNK/AKT signaling pathways.

## 1. Introduction

Colorectal cancer (CRC), also known as colon cancer or large bowel cancer, includes cancerous growths in the colon, rectum, anus, and appendix. CRC is the third most common type of cancer and the fourth leading cause of cancer-related death worldwide [1]. Treatments such as surgical excision, chemotherapy using cytotoxic drugs, and radiotherapy constitute the major current therapeutic regimens for colon cancer [2,3]. However, these therapeutic possibilities are only moderately successful for late-stage cancers and produce harmful side effects such as high toxicity or the increase of drug resistance and of the problems associated with metastasis. Therefore, novel therapeutic agents that target specific molecular signaling pathways in order to arrest CRC growth and metastasis are needed.

Traditionally, the search for novel drugs has largely relied on natural products (NPs) [4] and the identification of antitumor constituents from plants has been essential for advancing the chemotherapy of cancer [5]. During the last decades, the marine macro- and microorganisms have also been a rich and diversified source of biologically active molecules with a broad range of health beneficial effects, including anticancer properties [6,7]. Thus, numerous effective anticancer compounds have been discovered from natural sources and about 80% of chemotherapeutic agents developed so far for the treatment of cancer are based on NPs [8]. 

Within the marine environment, macroalgae are a rich reservoir of structurally diverse secondary metabolites, mostly belonging to the class of the terpenoids [9]. NPs of this class have exhibited a wide spectrum of antitumor activities [10] expressed by their capacity to regulate a variety of pathophysiological processes, such as proliferation, migration, invasion, apoptosis, and cell cycle in different types of tumor cells [11,12].

NPs of meroterpenoid type consisting of a terpenoid moiety attached to a toluquinone or a toluhydroquinone ring are widespread in brown algae of the family Sargassaceae [13]. In particular, algae of the genus *Cystoseira* have been the source of an array of this class of meroditerpenoids [14,15,16]. However, only a few of these compounds have been investigated for their biomedical properties, such as antioxidant, antibacterial, or cytotoxic activities [14]. Regarding the antitumor activity, the most recent reports have described the capacity of the meroterpenes cystoazorol A and cystoazorones A and B, isolated from *Cystoseira abies-marina*, to inhibit the growth of HeLa cells [17] and the cytotoxicity of cystoketal, obtained from *Cystoseira tamariscifolia*, towards HepG2 cells [18].

As a part of our research on bioactive metabolites from macroalgae, we observed that the extract of the seaweed *Cystoseira usneoides* exhibited significant activity as growth inhibitor of the colon cancer cells HT-29. In the present study, we investigated the antitumor properties of eight meroterpenoids isolated from the extract of *C. usneoides*. In particular, we examined the effects of these algal meroterpenoids (AMTs) on the viability, apoptosis, cell cycle, and motility of HT-29 colon cancer cells and we investigated the underlying mechanism of action. We found that several of the AMTs possess selectivity for colon cancer cells HT-29, showing lower toxicity on the normal colon cells CCD 841 CoN. Moreover, in in vitro assays, several of the tested AMTs increased apoptosis of HT29 colon cancer cells, caused cell cycle arrest at G2/M phase, and/or suppressed migration and invasion, which were associated with downregulation of the ERK (extracellular signal regulated kinase), JNK (c JUN N terminal kinase) and AKT (protein kinase B (PKB) signaling pathways. These results suggest the potential health benefits of the AMTs produced by the alga *C. usneoides* in colorectal cancer.

## 2. Materials and Methods 

### 2.1. Isolation and Identification of the Meroterpenoids 1-8 from the Alga C. usneoides

The collection of the alga, the isolation, and the structural characterization of the AMTs were performed as previously described [19]. Briefly, shade-dried samples of *C. usneoides* collected at the Gibraltar Strait were ground and extracted with acetone/methanol (MeOH). The resulting extract was subjected to column chromatography (CC) eluting with *n*-hexane/diethyl ether (Et_2_O) mixtures of increasing polarity, then Et_2_O, chloroform/MeOH mixtures, and finally MeOH. The fractions eluted with *n*-hexane /Et_2_O (30:70, *v/v*), Et_2_O, and chloroform/MeOH (95:5, *v/v*) were subjected to CC eluting with *n*-hexane/ethyl acetate (EtOAc) mixtures. Repeated separation of selected subfractions by normal phase HPLC using *n*-hexane/ EtOAc (60:40 and 50:50, *v/v*) or *n*-hexane/isopropanol (90/10, *v/v*) as eluents afforded eight pure compounds (**1**–**8**). CC was performed on silica gel (Merck KGaA, Darmstadt, Germany) and HPLC separations were performed on a LaChrom-Hitachi apparatus (Merck) equipped with LiChrospher Si-60 (250 × 10 mm, 10 μm) (Merck) and Luna Si (2) (250 × 4.6 mm, 5 μm) (Phenomenex, Torrance, CA, USA) columns, using an RI-71 differential refractometer or L-7400 UV detector (Merck). The structures of the isolated compounds were determined by using nuclear magnetic resonance (NMR) and mass spectrometry (MS). NMR spectra were recorded on an Agilent 500 spectrometer (Agilent Technologies, Santa Clara, CA, USA) using CD_3_OD or CDCl_3_ (Sigma-Aldrich, St. Louis, MO, USA) as solvent. HRMS spectra were obtained on a Waters SYNAPT G2 spectrometer (Waters, Milford, MA, USA). Fully assigned spectroscopic data of compounds **1**-**8** can be found in our previous paper [19].

### 2.2. Reagents for Bioactivity Assays

Sulforhodamine B (SRB), dimethylsulfoxide (DMSO), propidium iodide (PI), Tris-base, acetic acid, trichloroacetic acid (TCA), and RNase A were purchased from Sigma-Aldrich (Munich, Germany). Eagle’s minimal essential medium (EMEM) and fetal bovine serum (FBS) were from GIBCO (Grand Island, NY, USA). McCoy’s medium was from Sigma-Aldrich (Saint Louis, MO, USA). Phosphate buffer saline (PBS), streptomycine, penicillin, and trypsine-EDTA were from PAA-Laboratories GmbH (Pasching, Austria). The Annexin V-FITC Apoptosis Detection Kit was purchased from eBioscience, Thermo Fisher Scientific (Waltham, MA, USA). Trevigen’s Cultrex^®^ 96 Well Cell Invasion Assays was purchased from R&D Systems (Gymea, NSW, Australia). For Western blotting, primary and HRP-conjugated secondary antibodies anti-pERK1/2, anti-pJNK, and anti-pAKT were from Cell Signaling (Danvers, MA, USA), anti-β-actin from Santa Cruz Biotechnology (Dallas, TX, USA), and goat anti-mouse or -rabbit antibody from Dako Cytomation (Hamburg, Germany).

### 2.3. Cell Culture

HT-29 human colon carcinoma cells were obtained from the American Type Culture Collection (ATCC, Manassas, VA, USA). The cells were grown in McCoy’s medium supplemented with 10% FBS, 100U/mL penicillin, and 100 mg/mL streptomycin, and the culture was maintained in a humidified incubator at 37 °C under an atmosphere of 5% CO_2_. The normal human colonic epithelial cells CCD 841 CoN obtained from ATCC were grown in EMEM supplemented with 10% FBS, 100U/mL penicillin, and 100 mg/mL streptomycin, and the culture was maintained in a humidified incubator at 37 °C under an atmosphere of 5% CO_2_. 

### 2.4. Cell Viability Assay

Cell viability was quantified using the Sulforhodamine B assay described by Skehan et al. [20]. Cells were plated with a density of 5 × 10^3^ (HT-29) and 1 × 10^4^ (CCD 841 CoN) cells/well and allowed to attach at 37 °C in an atmosphere with 5% CO_2_. After 24 h, the AMTs dissolved in DMSO were added to the cells (final concentrations of 10, 20, 30, and 50 µg/mL and less than 0.05% of DMSO) and the plates were incubated for 72 h. Then, the cells were fixed by adding 50 μL of TCA (50%) to each well and the plates were maintained for 1 h at 4 °C. The plates were washed five times with deionized water, air-dried, and stained for 30 min at room temperature with 100 μL of 0.4% (*w/v*) SRB prepared in 1% (*v/v*) acetic acid. The plates were rinsed quickly five times with 1% acetic acid to remove unbound dye, followed by air-drying. The bound dye was solubilized in 2 mM Tris base (100 μL/well) for 5 min. Optical densities were read on a microplate reader (Spectrophotometer LabsystemsMultiskan EX, λ 492 nm). Three independent assays were conducted, each one in duplicate. A standard curve was constructed for each experiment and used for converting the measured optical density values into numbers of viable cells/well. The inhibition rate was used to evaluate the cell viability and its algorithm was the following formula: ((OD_control_ − OD_treatment_)/OD_control_) × 100; OD, optical density. The OD492 for control cells was taken as 100% viability. The half maximal inhibitory concentration (IC_50_) was calculated. The selectivity index (SI) of each compound was calculated as IC_50_ of the compound against the CCD 841 CoN normal cell line/IC_50_ of the same compound against the HT-29 cancer cell line.

### 2.5. Flow Cytometry Analysis for Apoptosis Induction Assay

Quantitative assessment of apoptosis was performed by flow cytometry using an Annexin V-FITC Apoptosis Detection Kit [21]. Briefly, 1 × 10^6^ HT-29 cells were seeded per well in six-well plates and incubated for 24 h, followed by the addition of the AMTs dissolved in DMSO (final concentrations of 10, 20, and 30 µg/mL for compounds 1, 2, 5, 6, 7, and 8, and 30, 60, and 90 µg/mL for compounds 3 and 4, and less than 0.05% DMSO) and incubation for 24 h. Curcumin (final concentrations of 50 and 75 μM) was used as positive control. The cells were then washed, harvested with trypsin, and centrifuged at 1500 rpm (5 min, 25 °C). The pellet was resuspended in 195 µL of 1 × Annexin buffer and then stained with 5 μL of Annexin V-FITC and 10 μL of PI for 10 min at room temperature, in the dark. To this mixture 200 μL of 1 × Annexin buffer was added before analysis using a Cytomics FC500 flow cytometer (Beckman Coulter, Indianapolis, IN, USA). Following a gating strategy, flow cytometry plots of size (FS) and complexity (SS) of the HT-29 cells were previously established for the elaboration of a measurement protocol. The normal healthy cells, early apoptosis, late apoptosis, and necrotic populations were represented by annexin V-negative/PI-negative population, annexin V-positive/PI-negative, annexin V-positive/PI-positive and annexin-negative/PI-positive cells, respectively. A total of 10,000 cellular events in each sample were analyzed using DML program.

### 2.6. Cell Cycle Analysis by Flow Cytometry

HT-29 cells (1 × 10^6^ cells/well) were seeded in six-well plates and incubated for 24 h, followed by treatment with AMTs dissolved in DMSO (final concentrations of 10, 20, and 30 µg/mL for compounds 1, 2, 5, 6, 7, and 8, and 30, 60, and 90 µg/mL for compounds 3 and 4, and always below 0.05% DMSO) and 24 h of additional incubation [22]. Colchicine (final concentration of 0.2 μg/mL) was used as positive control. The cells were then washed, harvested with trypsin, and centrifuged at 1500 rpm (5 min, 25 °C). The resulting pellet was fixed with ice-cold 70% ethanol (1 mL/10^6^ cells) and the samples were stored at −4°C overnight. After fixation, the cells were washed with PBS, stained with PBS containing 5 mg/mL of RNase A, and incubated for 48 h at 4 °C. Subsequently, 50 μL of 0.1 mg/mL of IP was added and incubated for 1 h at 4 °C. The relative DNA content per cell was analyzed using a Cytomics FC500 flow cytometer (Beckman Coulter, Indianapolis, IN, USA). The data acquisition was performed with the DML program and the analysis of the acquired data with the CXP Software (Beckman Coulter, Indianapolis, IN, USA).

### 2.7. Wound Migration Assay

For cell migration assay, HT-29 cells were seeded in six-well culture plates and allowed to grow to 80%–90% confluence for the experiment. After aspirating the medium, similar sized wounds were performed in the monolayer cells using a sterile micropipette tip. Wounded monolayer cells were washed three times with PBS to remove cell debris and then replaced with complete McCoy’s medium. HT-29 cells were treated with the AMTs (10 µg/mL for compounds 1, 2, 5, 6, 7, and 8, and 30 µg/mL for compounds 3 and 4) and incubated for 24h. For the image analysis of the effect of each treatment on cell migration, the wounded area was photographed immediately after scratching and after 24 h. The level of cell migration was determined using NIH Image software (Image J 1.44g, Wayne Rasband, USA) and then expressed as a percentage of wound closure area using the equation: Migration rate (%) = [(wound area at 0 h − wound area at 24 h)/wound area at 0 h] × 100%.

### 2.8. Cell Invasion Assay

The invasion activities were measured using Trevigen’s Cultrex^®^ 96 Well Cell Invasion Assays, as previously described [23]. Briefly, about 50 μL of of 1X basement membrane extract (BME) in coat buffer was added to each well. After incubation for 4 h at 37 °C in a 5% CO_2_ atmosphere, the HT-29 cells at 50,000 cells/50μL in serum free McCoy’s medium were added per well to the top chamber containing the tested compound (10 µg/mL for 1, 2, 5, 6, 7, and 8, and 30 µg/mL for compounds 3 and 4). About 150 μL of McCoy’s medium was then added to the lower chamber containing 10% FBS and penicillin/streptomycin as chemoattractants. Cells were allowed to migrate to the lower chamber in a humidified atmosphere containing 5% CO_2_ at 37 °C for 24 h. Afterwards, top and bottom chambers were aspirated and washed with washing buffer supplemented with the kit. About 100 μL of Cell Dissociation Solution/Calcein-AM was added to each bottom chamber well and incubated for 1 h at 37 °C in CO_2_ incubator. The cells internalized calcein-AM and the intracellular esterases cleaved the AM moiety to generate free calcein. Fluorescence of the samples was determined at 485 nm excitation, 520 nm emission, using ELISA plate reader (BioTek Instruments, Winooski, VT, USA). The number of cells that had invaded through the BME coat was calculated using a standard curve. Results were expressed as percentage of invasion (%) compared to the control.

### 2.9. Preparation of Cell Lysates

Cells were cultured to 80% confluence at 37 °C. After 24 h of treatment with the compounds (10 and 20 µg/mL for compounds 1, 2, 5, 6, 7, and 8, and 30 and 60 µg/mL for compounds 3 and 4), HT-29 cells were rinsed twice with ice-cold PBS, and then lysed immediately in lysis buffer (250 mM NaCl, 50 mM Tris (pH 7.5), 0.5 mM EDTA, 5 mM EGTA, 8 mM MgCl_2_, 1 mM PMSF, 0.01 mg/mL pepstatin A, 0.01 mg/mL leupeptin, 0.01 mg/mL aprotinin, 1% Triton X-100). After centrifugation at 12,000 g for 3 min at 4°C to separate the cellular debris, the supernatant was collected and stored at −80°C until use. The protein concentrations were determined using Bio-Rad Protein Assay (BioRad, Richmond, CA, USA), according to the manufacturer’s instructions.

### 2.10. Western Blot Analysis

Equal protein content (50 μg) samples of cell lysates were separated on 10% SDS–polyacrylamide gel and transferred to polyvinylidene difluoride membranes (Hybond-P, Amershan Biosciences, Little Chalfont, UK). The membranes were blocked with 5% (*w/v*) non-fat dry milk in Tris-buffered saline containing 0.1% Tween-20 buffer (pH 7.4) (TBST) for 1 h at room temperature and incubated with agitation with specific antibodies: anti-pERK1/2 (Cell Signaling; 1:1000), anti-pJNK (Cell Signaling; 1:1000), anti-pAKT (Cell Signaling; 1:1000), anti-β-actin (Sigma-Aldrich; 1:500). Membranes were incubated overnight at 4°C with gentle shaking. The secondary antibody was a peroxidase-conjugated goat anti-mouse or -rabbit antibody (1:10,000; DakoCytomation, Carpinteria, CA, USA). After washing the membrane three times in TBST buffer (10 min), the signals were detected using an enhanced chemiluminescence light-detecting kit (Super-Signal West Pico Chemiluminescent Substrate, Pierce, IL, USA), according to the manufacturer’s instructions and exposed to an X-ray film (GE Healthcare Ltd., Amersham, UK). The protein band densities were analyzed and quantified with a Scientific Imaging Systems (Biophotonics ImageJ Analysis Software, National Institute of Mental Health, Bethesda, MD, USA). β-actin was used to confirm the equal loading and transfer of proteins.

### 2.11. Statistical Analysis

The results were means ± standard error (SE) of three duplicates, and expressed as percentage respect to the control group (100%). In the flow cytometer studies, results were expressed as percentages of cells in Sub-G0, G0-G1, S, and G2/M in the cell cycle analysis, or percentages of cells in the different forms of apoptosis. Data were evaluated with GraphPad Prism^®^ Version 5.00 software. Differences between two groups were analyzed by the Student’s *t*-test. Difference with *p* < 0.05 (*), *p* < 0.01 (**), or *p* < 0.001 (***) were considered statistically significant.

## 3. Results

**Figure 1:**
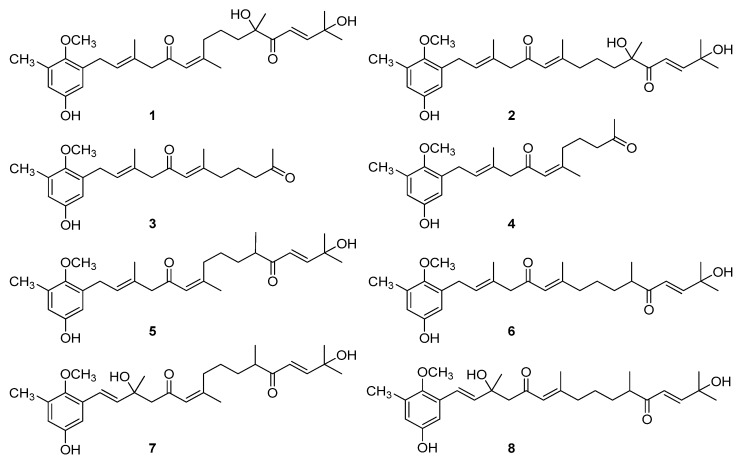
Chemical structures of the meroterpenes (algal meroterpenoids—AMTs) from the alga *Cystoseira usneoides* subjected to anticancer studies: usneoidone Z (**1**), 11-hydroxy-1′-O-methylamentadione (**2**), cystomexicone B (**3**), cystomexicone A (**4**), 6-cis-amentadione-1′-methyl ether (**5**), amentadione-1′-methyl ether (**6**), cystodione A (**7**), and cystodione B (**8**).

The AMTs usneoidone Z (**1**), 11-hydroxy-1′-O-methylamentadione (**2**), cystomexicone B (**3**), cystomexicone A (**4**), 6-cis-amentadione-1′-methyl ether (**5**), amentadione-1′-methyl ether (**6**), cystodione A (**7**), and cystodione B (**8**) (Figure 1), isolated from the alga *C. usneoides*, were investigated for their anticancer activity.

### 3.1. The AMTs 1-8 Inhibit Cell Proliferation in Human Colon Adenocarcinoma Cells HT-29

**Figure 2:**
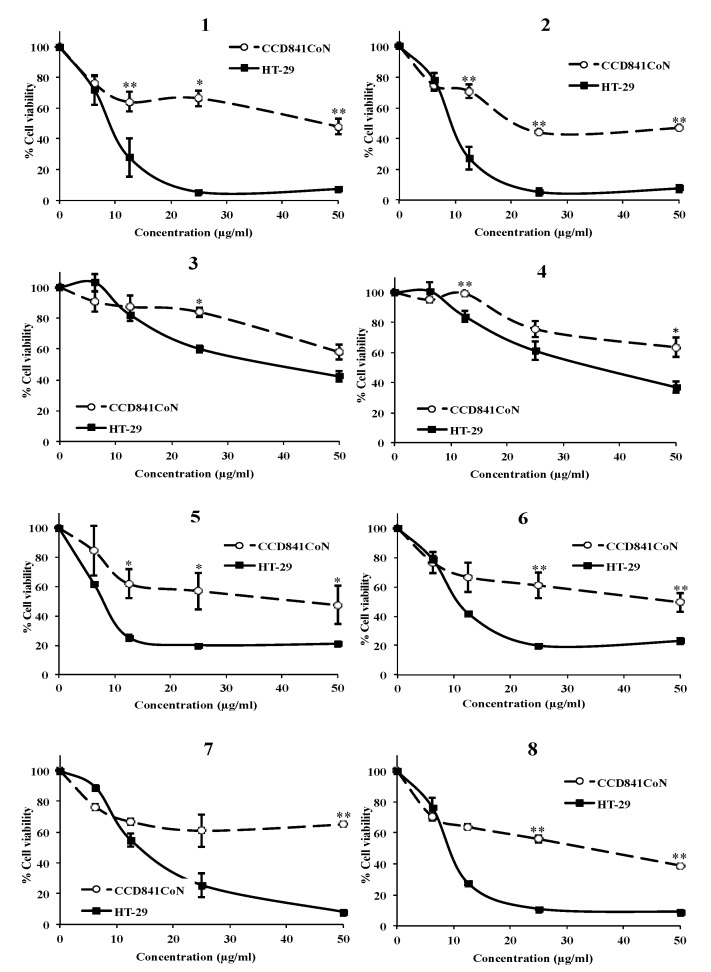
Effect of meroterpenes (AMTs) **1**-**8** at different concentrations on the viability of both HT-29 colon cancer cells and normal colonic epithelial cells CCD 841 CoN after 72 h of treatment. Results obtained by the sulforhodamine B (SRB) assay are reported as the percentage of viable cells (% cell viability). Data represent mean ± SE from three independent experiments. * *p* < 0.05 and ** *p* < 0.01 compared with the untreated group.

The ability of the compounds **1**-**8** at different concentrations to inhibit the viability of cancer and non-cancer colon cells (HT-29 and CCD 841 CoN, respectively) was examined by the SRB assay. All compounds caused a dose-dependent decrease in cell survival for both cancer and non-cancer cells, although at different extents (Figure 2). Usneoidone Z (**1**) and 6-*cis*-amentadione-1′-methyl ether (**5**) showed the strongest growth inhibitory activity against colon cancer cells HT-29 (IC_50_ 8.81 and 7.83 µg/mL, respectively), while the effects of both compounds were much lower towards the normal colon cells CCD 841 CoN (IC_50_ 46.41 and 40.97 µg/mL, respectively) (Table 1). The meroditerpenes **2**, **6**, **7**, and **8** also induced strong decreases of the viability of HT-29 cells, greater than those observed for CCD 841 CoN cells (IC_50_, 9.14, 10.72, 14.00, and 9.14 µg/mL for HT-29 cells and IC_50_ 21.41, 48.38, >50, and 31.88 µg/mL for CCD 841 CoN cells, respectively). Compounds **3** and 4 were the less cytotoxic towards both the tumor and the normal cells.

According to the literature, compounds with selectivity index (SI) values greater than 3 are considered highly selective [24], although other authors consider that compounds with a SI higher than or equal to 2.0 are also interesting [25]. Treatments with the AMTs **1** and **5** afforded selectivity indexes higher than 5 (SI = 5.26 and 5.23, respectively), indicating that these compounds are five times more toxic towards the cancer cells than towards the non-cancer cells (Table 1). The AMTs **6**, **7**, and **8** were also highly selective for cancer cells, affording SI higher than 3 (4.51, >3.57, and 3.51, respectively). However, the selectivity indexes observed for **2**, **3**, and **4** were more moderated (2.34, >1.45, and 1.68, respectively).

### 3.2. The AMTs 11-hydroxy-1′-O-methylamentadione (2), cystomexicone B (3), and 6-cis-amentadione-1′-methyl ether (5) Induce Apoptosis of HT-29 Colon Cancer Cells

**Figure 3:**
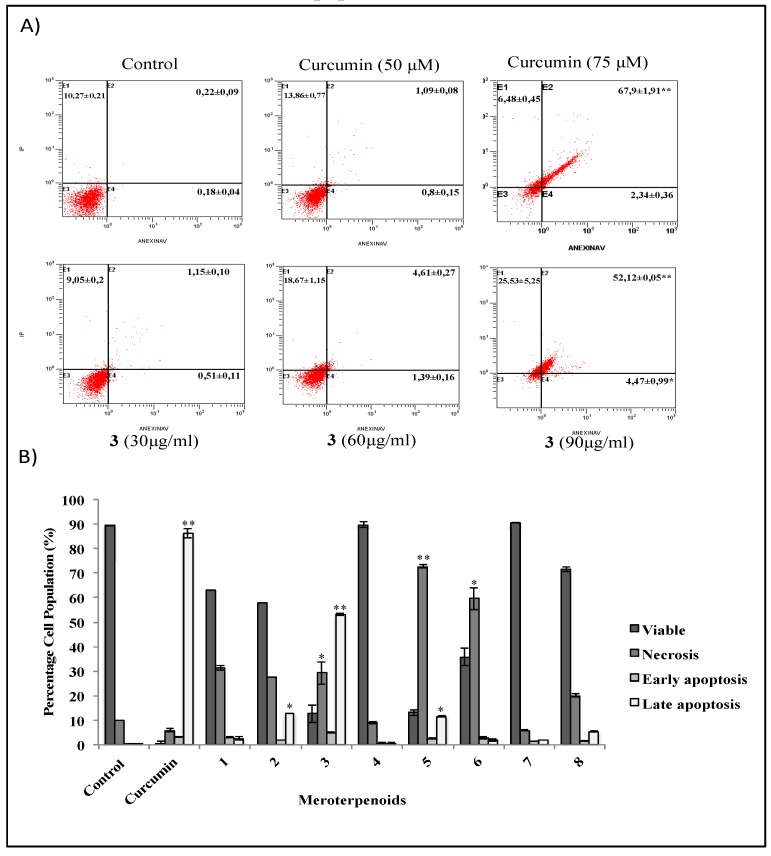
Apoptosis rates by flow cytometry of HT-29 colon cancer cells treated with the meroterpenes (AMTs) **1**-**8** for 24 h. Negative control cells received no treatment. Positive control received 50 and 75 μM of curcumin. (**A**) Representative flow cytometry histograms showing the percentage of HT-29 cells undergoing early and late apoptosis after treatment with 30, 60, and 90 μg/mL of AMT **3**. The abbreviations are necrosis (E1), early apoptosis (E2), viable (E3), and late apoptosis (E4). (**B**) Bar charts illustrate the percentage of viable, necrotic, early, and late apoptosis cells treated for 24 h with 28 μg/mL of curcumin (positive control), 30 μg/mL of **1**, **2**, **5**, **6**, **7**, and **8**, and 90 μg/mL of **3** and **4**. Data represent mean ± SE from three independent experiments. Significant differences from control group: * *p* < 0.05 and ** *p* < 0.01.

Flow cytometry analysis by annexin V/PI staining was performed in order to investigate the induction of apoptosis in HT-29 cells by AMTs **1**-**8**. Compounds **1**, **2**, **5**, **6**, **7**, and **8** were tested at concentrations of 10, 20, and 30 μg/mL, and compounds **3** and **4** were tested at 30, 60, and 90 μg/mL. The percentages of viable, early apoptotic, late apoptotic, and necrotic cells after 24 h of treatment with the AMTs **1**-**8** are shown in Figure 3. Significant differences were observed between control and treated cells. A total of 24 h after treatment, 89.34% of vehicle alone-treated HT-29 cells were viable (Annexin V-PI-), 0.18% were early apoptotic cells (Annexin V+PI-), and 0.22% were late apoptotic (Annexin V+PI+). In contrast, HT-29 cells treated for 24 h with cystomexicone B (**3**), showed a progressive induction of the apoptosis process (Figure 3A), with a significant increase at the dose of 90 μg/mL, both in the early (4.47%) and in the late (52.12%) apoptotic cells (total percentage of apoptotic cells, 56.6%). As shown in Figure 3B, the AMT **2** also caused a significant, although more moderated, apoptotic effect (57.7% of viable cells, 1.94 % early apoptotic, and 12.94 % late apoptotic) (Figure 3B). Compound **5** induced a percentage of apoptosis similar to **2**, but the most prominent effect was necrosis (72.75%). In this line, although cell growth was not affected by the AMTs **1**, **6**, and **8** at concentrations up to 20 μg/mL, at the higher concentration of 30 μg/mL a strong necrotic effect was detected, with 31.22%, 59.55%, and 20.12% of dead cells, respectively. After 24 h of treatment, the AMTs **4** and **7** did not induce neither apoptosis nor death of HT-29 cells.

### 3.3. Effects of the AMTs 1-8 on Cell Cycle Arrest in HT-29 Cells

**Figure 4:**
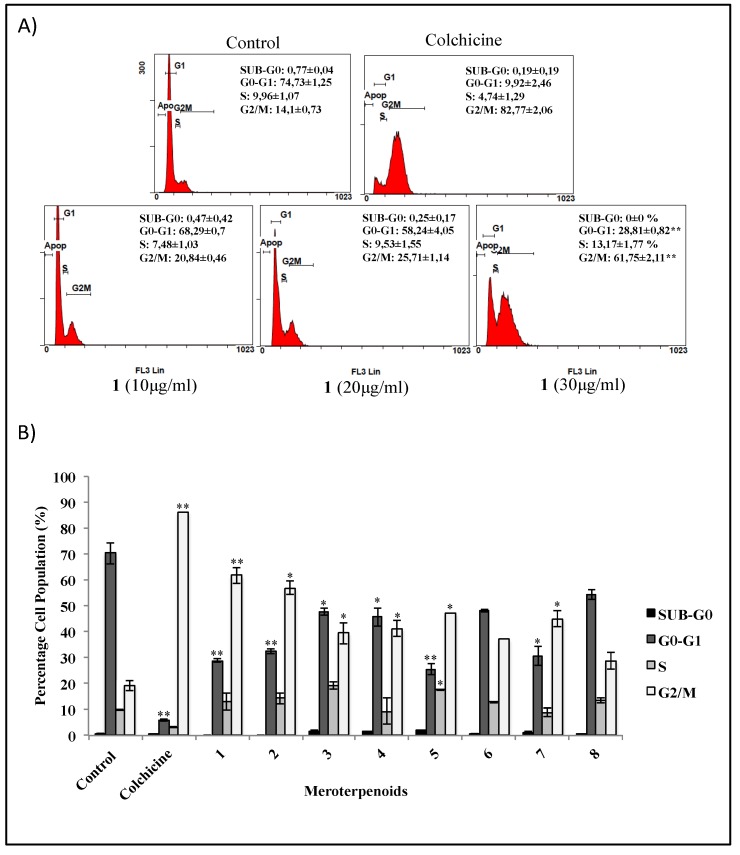
Flow cytometry analysis of cell cycle arrest in colon cancer cells HT-29 treated for 24 h with the meroterpenes (AMTs) **1**-**8**. Negative control cells received no treatment. Positive control received 0.2 μg/mL of colchicine. (**A**) Representative flow cytometry histograms, for cell cycle phase (percentages in Sub-G0, G0-G1, S, and G2/M,) showing HT-29 cells treated with 10, 20, and 30 μg/mL of compound **1**. (**B**) Similarly, bar charts for HT-29 cells treated with 30 μg/mL of **1**, **2**, **5**, **6**, **7**, and **8**, and 90 μg/mL of **3** and **4**. Data represent mean ± SE from three independent experiments. Significant differences to control group: * *p* < 0.05 and ** *p* < 0.01.

In an attempt to explore the effects of the AMTs **1**-**8** on the cell cycle progression of colon carcinoma cells HT-29, the cell cycle was analyzed by flow cytometry. The effects of increasing concentrations of usneoidone Z (**1**) on HT-29 cell progression through G0/G1-, S-, and G2/M-phases are shown in Figure 4A. This compound was the most active among the tested AMTs and increasing concentrations (10, 20, 30 μg/mL) resulted both in a significant cell cycle arrest in the G2/M (*p* < 0.01) and in the reduction of the number of cells in the G0/G1 phase (*p* < 0.01). The accumulation of cells at the G2/M phase was also significant with the AMTs **2**, **3**, **4**, **5**, and **7** (*p* < 0.05) and it was correlated with a subsequent significant decrease of cells in the G0/G1-phase (Figure 4B). Compounds **6** and **8** showed the same tendency in cell cycle progression but the changes were not significant.

### 3.4. Effects of the AMTs **1**-**8** on the Migration and Invasion of HT-29 Cells

**Figure 5:**
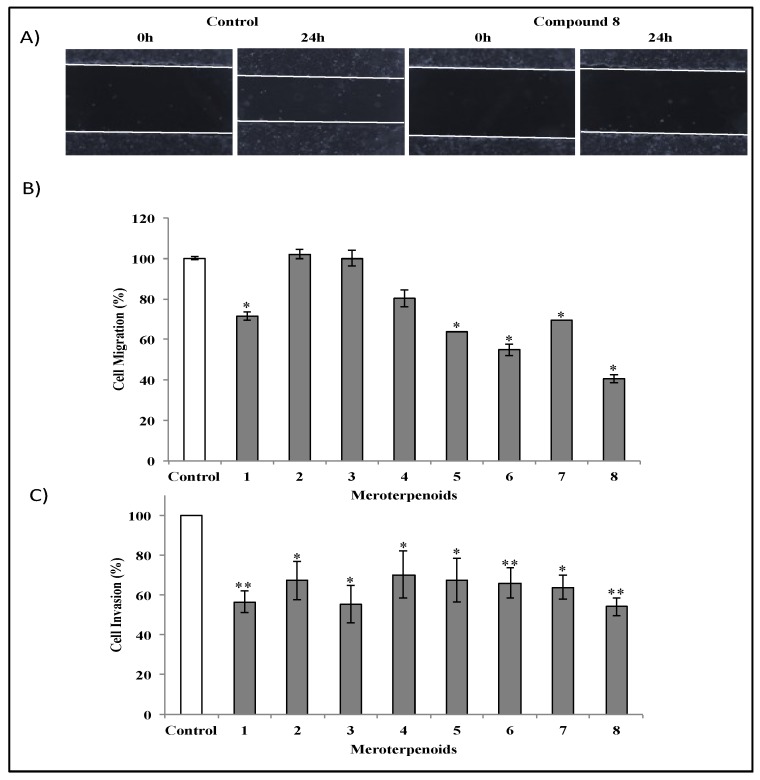
Effects of meroterpenes (AMTs) **1-8** on migration and invasion of HT-29 cells. (**A**) Seeded colon cancer cells were wounded and imaged (0 h). Untreated HT-29 cells and cells treated with compound **8** (10 μg/mL) were incubated for 24 h. (**B**) Bar charts show migrated HT-29 cells after treatment for 24 h with **1**, **2**, **5**, **6**, **7**, and **8** at 10 μg/mL and with **3** and **4** at 30 μg/mL. (**C**) Anti-invasive activity of AMTs against HT-29 cells measured using a Cultrex assay kit. HT-29 cells were untreated or treated for 24 h with **1**, **2**, **5**, **6**, **7**, and **8** at 10 μg/mL and with **3** and **4** at 30 μg/mL. The result of control groups was set as 100% activity and test groups were compared to this value. The data represent the means ± SE of three independent experiments. Significant differences to control group: * *p* < 0.05 and ** *p* < 0.01.

Cell migration is a measure of the metastatic potential of cancer cells. To examine whether the AMTs **1**-**8** had any inhibitory effect on cell migration process, HT-29 cells were incubated for 24 h in the absence or presence of the compounds (10 μg/mL) in a wound-healing assay (Figure 5). According to the quantitative assessment, cystodione B (**8**) was the most active (Figure 5), causing at 10 µg/mL 59.1% inhibition of cell migration after 24 h (*p* < 0.05). As shown in Figure 5B, HT-29 cell migration to the wounded area was also significantly inhibited by 28.42%, 36.19%, 45.26%, and 30.72% (*p* < 0.05) in the presence of 10 µg/mL of compounds **1**, **5**, **6**, and **7**, respectively. However, compounds **2**, **3**, and **4** showed no significant effects on cell migration. Overall, these data demonstrated that most of the merterpenoids of *C. usneoides* have significant inhibitory effects on the migration of HT-29 cells. Another important characteristic of metastasis is the invasive ability of cancer cells. To determine the inhibitory effect of the AMTs **1**-**8** on the invasion of HT-29 cells, we used Cultrex^®^ 96 well basement membrane extract (BME) cell invasion assay kit. The range of inhibition caused by the AMTs at 10 μg/mL was 30%–45% (Figure 5C) when cells were incubated for 24 h. Among the tested compounds, usneoidone Z (**1**), cystomexicone B (**3**), and cystodione B (**8**), were the most active inhibiting cell invasion by more than 40% (*p* < 0.01 for compounds **1** and **8**, and *p* < 0.05 for compound **3**). These results demonstrated that all the tested AMTs can directly inhibit the invasive potential of colon cancer cells, thus indicating the interesting anticancer potential of these NPs.

### 3.5. The AMTs **1**-**8** Inhibit Phosphorylation of ERK, JNK, and AKT

**Figure 6:**
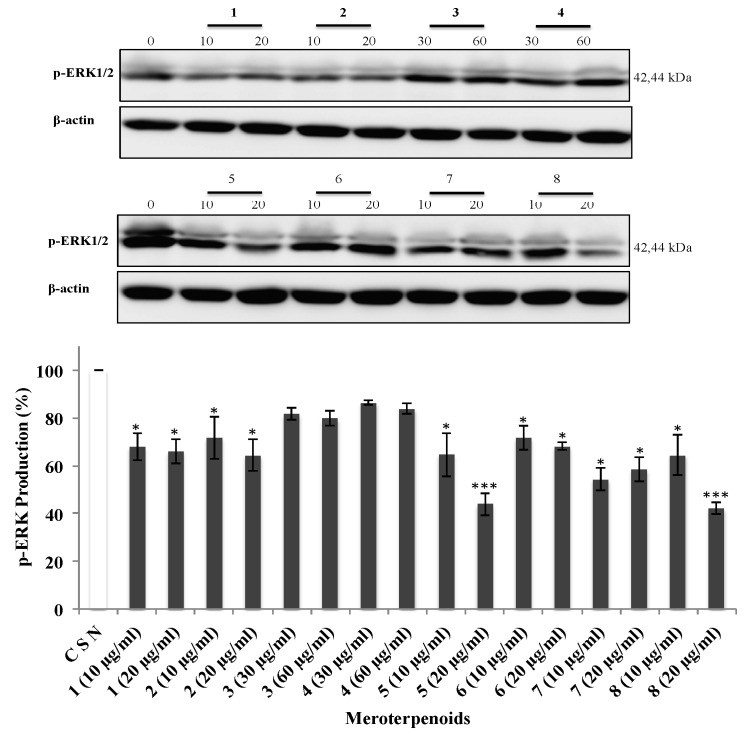
Effect of meroterpenes (AMTs) **1-8** on the activation of extracellular signal-regulated kinase (ERK) by measuring the expression levels of the phosphorylated form (p-ERK) in HT-29 cells. The cells were treated for 24 h with the compounds (10 and 20 μg/mL for **1**, **2**, **5**, **6**, **7**, and **8**; 30 and 60 μg/mL for **3** and **4**). The levels of p-ERK were measured by Western blot analysis and quantified with Image J analysis software. The result of control groups was set as 100% activity and test groups were compared to this value. The data shown are the means ± SE of three independent experiments. Significant differences from control group: * *p* < 0.05 and *** *p* < 0.001.

**Figure 7:**
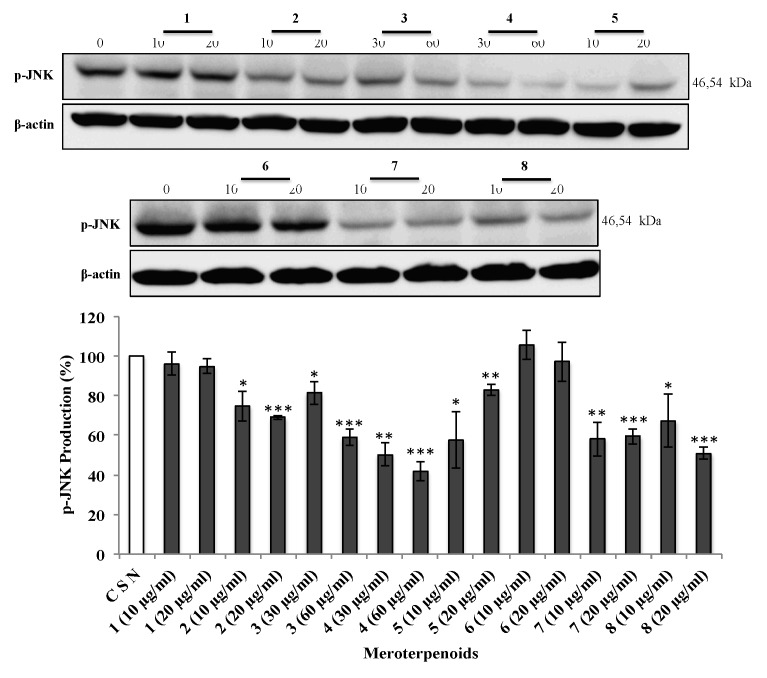
Effect of meroterpenes (AMTs) **1-8** on the activation of c-JUN N-terminal kinase (JNK) by measuring expression levels of the phosphorylated form (p-JNK) in HT-29 cells. The cells were treated for 24 h with the compounds (10 and 20 μg/mL for **1**, **2**, **5**, **6**, **7**, and **8**; 30 and 60 μg/mL for **3** and **4**). The levels of p-JNK were measured by Western blot analysis and quantified with Image J analysis software. The result of control groups was set as 100% activity and test groups were compared to this value. The data shown are the means ± SE of three independent experiments. Significant differences from control group: * *p* < 0.05, ** *p* < 0.01, and *** *p* < 0.001.

**Figure 8:**
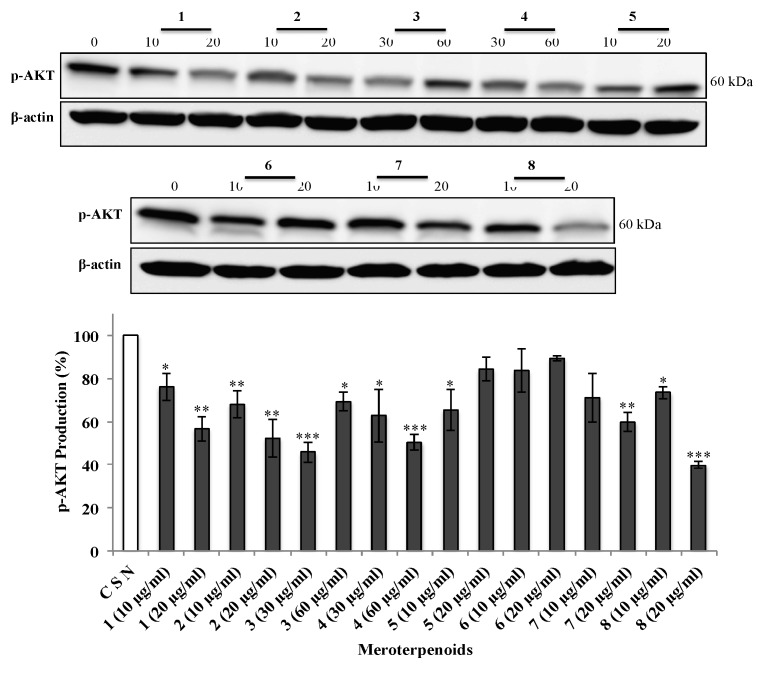
Effect of meroterpenes (AMTs) **1-8** on the activation of protein kinase B (AKT) by measuring expression levels of the phosphorylated form (p-AKT) in HT-29 cells. The cells were treated for 24 h with the compounds (10 and 20 μg/mL for **1**, **2**, **5**, **6**, **7**, and **8**, and 30 and 60 μg/mL for **3** and **4)**. The levels of p-AKT were measured by Western blot analysis and quantified with Image J analysis software. The result of control groups was set as 100% activity and test groups were compared to this value. The data shown are the means ± SE of three independent experiments. Significant difference from control group, * *p* < 0.05, ** *p* < 0.01, and *** *p* < 0.001.

Since the previous findings showed that the AMTs **1-8** significantly inhibit migration and/or invasion of HT-29 cells, the underlying mechanism was further investigated, in particular the cell signaling pathways. Various studies suggest that MAPKs (ERK 1/2, JNK 1/2, and p38) and AKT are important players in cancer cell migration and invasion [26,27]. In view of this evidence, the effects of the AMTs **1-8** on the phosphorylation of ERK1/2, JNK, and AKT were examined on HT-29 cells. The cancer cells were treated for 24 h with various concentrations of the AMTs and the phosphorylation of ERK1/2, JNK, and AKT were measured by Western blot analysis. The compounds **1**, **2**, **5**, **6**, **7**, and **8** significantly reduced the p-ERK1/2 (Figure 6) compared to the control group (CSN), with the maximum inhibitory effect observed in cells treated with **5** and **8** at the highest concentration (20 µg/mL). As shown in Figure 7, all the tested AMTs, except for the meroditerpenes **1** and **6**, significantly inhibited phospho-JNK when compared with that of the control. The compound that had the maximum inhibitory effect was **4** at the highest concentration (60 µg/mL). The data also showed that the expression of p-AKT protein was significantly downregulated in cells treated with **1**, **2**, **3**, **4**, **7**, and **8** (Figure 8), and there was no significant reduction of p-AKT production on cells treated with the AMTs **5** and **6**. Based on these results, the mechanism for the inhibition of the metastatic activity on HT29 cells caused by the AMTs **1**-**8** could be partly explained by inducing the suppressions of ERK1/2, JNK, or AKT pathways.

## 4. Discussion

In this study various assays were used to investigate the antitumor effects on human colon cancer HT-29 cells caused by eight AMTs isolated from the bioactive extract of the alga *C. usneoides*. We first determined that the AMTs **1**, **2**, **5**, **6**, **7**, and **8**, exhibited growth inhibitory activity against HT-29 cells with IC_50_ values in the range 7.8–14.0 μg/mL while compounds **3** and **4** were less potent (IC_50_ = 36.9 and 34.3 μg/mL, respectively). Interestingly, all the AMTs showed IC_50_ values significantly higher against the non-cancer cells CCD 841 CoN, with a selectivity index of 5.26 for compound **1** and 5.23 for **5**. We also investigated if the growth suppression induced by the AMTs **1**-**8** is mediated by apoptosis and cell cycle arrest. Our results showed that the compounds isolated from *C. usneoides* (except for **6** and **8**) induced significant anticancer effects against HT-29 cells through G2/M cell progression arrest, although only compounds **2**, **3**, and **5** produced apoptosis of the colon cancer cells. Moreover, we showed the significant inhibitory effects of most of the tested AMTs on migration and invasion of the human colon cancer HT-29 cell line. This is the first report on the antitumor activity of the AMTs **1-8** against colon cancer cells. Moreover, among the tested compounds, there was only previous antitumor data for usneoidone Z (**1**) [28]. From the point of view of the structure–activity relationships the results of growth inhibition of HT-29 cells evidence the higher activity of compounds displaying a terpenoid chain of 20 carbon atoms (**1**, **2**, **5**, **6**, **7**, and **8**) upon comparison with those with a chain of 14 carbon atoms (**3** and **4**). On the other hand, the similar IC_50_ values shown by compounds **1**, **2**, **5**, **6**, and **8** suggest that other structural features such as the configuration of the double bond at C-6, C-7 and the presence of an additional hydroxy group at C-3 or C-11, do not affect significantly to the growth inhibitory activity on HT-29 cells. There is little previous data on the growth inhibitory activity of AMTs against colon cancer cells HT-29. In particular, it has recently been reported that the AMTs sargachromanols J and R, from the alga *Sargassum siliquastrum* [29], and zonaquinone acetate, from *Stypopodium zonale* [30], inhibited the growth of HT-29 cells with IC_50_ values of 29.3 μg/mL, 3.4 μg/mL, and 17.3 μM (7.8 μg/mL), respectively, which are comparable to those obtained in our study. In the present study, we were able to demonstrate that the antitumor activity of the AMT cystomexicone B (**3**) is exerted through induction of apoptosis. This finding was consistent with previous reports that demonstrated the apoptotic effects caused by various AMTs [29,30]. In particular, the AMT tuberatolide B, isolated from the alga *Sargassum macrocarpum* [31], has been shown to inhibit the viability of various cancer cell lines, including breast cancer (MDA-MB-231), lung cancer (A549), and colon cancer (HCT116), by inducing apoptotic cell death, and sargachromanol E, from *Sargassum siliquastrum*, has been reported to induce apoptosis in the colon cancer cell line HL-60 [32]. On the other hand, in the last years a variety of algal terpenoids have been reported to induce apoptosis in several cancer cells, including HT-29 [33], Jurkat leukemic cells [34], melanoma B16F10 cells [35,36], and human TNBC cells [37].

Cancer is a complex pathology where the cells undergo different transformations, among which uncontrolled cell division stands out. Cell cycle deregulation is the hallmark of cancer progression and the control of the cell cycle helps to regulate cell growth. This is one of the most critical alterations during tumor progression and plays an important role in apoptosis [38]. The G2/M checkpoint is a known target for cell cycle inhibition [39], which marks a barrier before entry into M phase [40]; in this way it is critical to prevent progression through mitosis when cells progress into G2 with an unrepaired DNA during the previous S or G1 phases, or when they possess incompletely replicated chromatin from S phase [41]. A consequence is that cells with DNA damage can initiate an apoptotic program, that leads to the phenotypic manifestation of mitotic failed during the metaphase [42]. In this study, we demonstrated that the growth inhibitory effect of the AMTs **1**, **2**, **3**, **4**, **5**, and **7** on HT-29 cancer cells is associated with a G2/M arrest and cell cycle progression. These results are in line with an earlier report that demonstrated that the algal halogenated monoterpene mertensene induced similar response with G2/M arrest from HT-29 cells [33]. However, other algal terpenes have been described to induce cell cycle arrest in G1 phase in different types of cell lines [36,37]. Cancer metastasis is a leading cause of death in cancer patients. The migration and invasion of cancer cells allow them to detach from the primary tumor to the surrounding tissues and colonize the target organs [43]. Interestingly, the present study demonstrated that the treatment of cells with the AMTs **1**-**8** decreases migration and/or invasion of the colon cancer cells.

The MAPK signaling pathway that consists of extracellular signal-related kinase 1 and 2 (ERK1/2), c-JUN N-terminal kinase/stress activated protein kinase (JNK/SAPK), and p38 [44], is involved in cell survival, cell-cycle progression, programmed cell death, and metastasis of cancer cells [45]. In colorectal carcinoma, the MAPK pathway is aberrantly activated [46] and, therefore, the inhibition of this pathway is a potential therapeutic approach [45]. It has been shown that the ERK promotes growth, differentiation, and proliferation of cancer cells [47]. It has also been reported that transient ERK activation might be linked to cellular proliferation while strong and persistent activation may lead to programmed cell death [48]. ERK promotes either intrinsic or extrinsic apoptotic pathways by induction of mitochondrial cytochrome c release or caspase-8 activation [49]. The ERK pathway not only participates in the regulation of apoptosis, but also controls G2/M cell cycle phase. ERK has been reported to regulate cyclin B1 transcriptional induction and also controls the assembly of cyclin-CDK complex via the CDK translocation [50,51], which play pivotal roles in regulating cell cycle progression. Moreover, ERK1/2 regulates focal adhesion and cytoskeletal reorganization via the phosphorylations of specific cytoskeletal and focal adhesion proteins, including paxillin, FAK, and myosin light chain kinase, which are crucial signaling components to control cell migration, invasion, and cell cycle progression [52,53]. In this study, we examined the effect of compounds **1-8** on the ERK pathway. A decreased protein expression of phosphorylated ERK was observed in HT-29 cells treated with compounds **1**, **2**, **5**, **6**, **7**, and **8**, which support the crucial role of ERK in the regulation of proliferation, cycle progression, and metastasis processes in colon cancer cells. Activation of JNK has been involved in the regulation of various cellular processes, including cell survival, proliferation, differentiation, and cell death [54]. However, few papers have been published regarding to the potential role of JNK in the cell cycle. Mingo-Sion et al. [55] reported that the induced G2/M arrest may be due to the inability of some cells to sustain p21^Cip1/Waf1^, a JNK substrate, in the absence of JNK activity. When p21^Cip1/Waf1^ expression is increased in response to DNA damage, cyclin B/Cdk1 kinase activity is inhibited causing G2/M phases arrest [56]. In this study, we found that compounds **2**, **3**, **4**, **5**, **7**, and **8** inhibit cell proliferation through inducing a G2/M phase arrest in HT-29 cells via the JNK pathway. Regarding programed cell death, JNK plays an active role in the regulation of both the intrinsic and extrinsic apoptotic pathways [57]. These findings suggest that p-JNK may be involved in the activation of cell apoptosis after the treatment with compound **3**. JNK also plays a crucial role in cell migration and invasion. The oncogenic functions of JNK are particularly based on its ability to phosphorylate c-Jun and to activate transcriptional factor Activator Protein-1 (AP-1). Matrix metalloproteinases (MMPs), a key role in degrading the basement membrane, have an AP-1 consensus sequence that regulates tumor progression by enhancing tumor-induced angiogenesis and destroying local tissue architecture and basement membranes to allow tumor invasion and metastasis [58]. The AMTs showing anti-migration (**5**, **7**, and **8**) and anti-invasion (**2**, **3**, **4**, **5**, **7**, and **8**) activities also decreased phosphorylation of JNK in colon cancer cells, suggesting that these compounds may have efficacy in the prevention of the metastasis of colon cancer cells.

Alteration of the PI-3K/AKT pathway has been detected during tumor formation in numerous cancers, including colorectal cancer [59]. Many studies have reported that PI3K activation stimulates the downstream target AKT, which plays various and important roles in regulating cell proliferation, cell cycle, apoptosis, and cell invasion [46,60,61]. The AKT pathway has been shown to be involved in the cell cycle progression by downregulating Cdk1 and Cyclin B1 expression, both of which can ultimately lead to the arrest of G2/M transition [62]. On the other hand, AKT modulates apoptosis signaling by inducing expression of multiple pro-apoptotic members of the Bcl2-family of mitochondria-targeting proteins. These pro-apoptotic proteins translocate in the mitochondria leading to caspase activation thus leading to apoptosis [63]. AKT is also known to regulate the expression of FAK (focal adhesion kinase) proteins mediating colorectal cancer metastasis. In response to extracellular pressure, AKT and FAK bind directly, thus phosphorylating AKT at three serine residues. The phosphorylation of the three serine residues consequently phosphorylate the tyrosine residue (Tyr397) thus activating it. It therefore induces cell adhesion by increasing the binding of integrins to matrix, which finally lead to increased metastasis [64]. Hence, inhibiting AKT may be an important therapeutic target for regulating cell cycle progression, apoptosis, and preventing cancer metastasis. In the present study, we found that the AMTs **1**, **2**, **3**, **4**, **7**, and **8** reduce the protein levels of p-AKT in HT-29 cells, indicating the role of these AMTs in the downregulation of proliferation, cell cycle, apoptosis, and metastasis in colon cancer cells through the regulation of MAPK and AKT pathways. These results are in line with a previous study where the algal halogenated monoterpene mertensene was shown to induce G2/M cell cycle arrest and apoptosis in human colon adenocarcinoma HT-29, through the modulation of ERK-1/-2 and AKT signaling [33]. Nonetheless, some studies on the mechanism of action of other algal terpenoids have also demonstrated the intervention of other signaling pathways. For example, the sesquiterpene guai-2-en-10α-ol, from *Ulva fasciata*, was reported to induce apoptosis and cell cycle arrest in G1 phase of triple-negative breast cancer (TNBC) cell line (MDA MB-231) via regulation of EGFR/PI3 K/Akt pathway [37] and laurinterol, from *Laurencia okamurae*, showed anticancer activity against melanoma cells (B16F1) through the p53-dependent pathway [65].

## 5. Conclusions

In summary, the present study demonstrates for the first time that various AMTs obtained from the alga *C. usneoides* inhibit in vitro the proliferation of colon cancer cells, while being significantly less cytotoxic against normal cells, induce cell cycle arrest, and decrease migration and invasion of HT-29 cells. Moreover, with cystomexicone B (**3**), apoptosis in HT-29 cells was detected. Our results provide proof that the tested AMTs promote anticancer effects through downregulation of signaling pathways by ERK, JNK, and/or AKT. On the basis of the activity observed for the AMTs **1**, **2**, **5**, **7**, and **8** in most of the assays performed in this study, these AMTs could be promising as agents for the prevention and treatment of colon cancer, although the potential of these NPs to act as chemopreventive and therapeutic agents for colorectal carcinomas needs to be previously evaluated in animal models.

## Figures and Tables

**Table 1:** IC_50_ values (μg/mL) obtained for meroterpenes (AMTs) **1**-**8** against the colon cancer cells HT-29 and the normal colon cells CCD 841 CoN after 72h of treatment (data are means ± SE of three experiments). SI = IC_50_ value for normal cells/ IC_50_ value for cancer cells.

	Cell Lines		Selectivity Index (SI)
Compound	CCD841 CoN	HT-29	
**1**	46.41 ± 3.87	8.81 ± 1.55	5.26
**2**	21.41 ± 2.50	9.14 ± 0.23	2.34
**3**	>50	34.34 ± 1.81	>1.45
**4**	61.86 ± 2.37	36.86 ± 2.86	1.68
**5**	40.97 ± 9.80	7.83 ± 0.05	5.23
**6**	48.38 ± 3.84	10.72 ± 0.27	4.51
**7**	>50	14.00 ± 1.33	>3.57
**8**	31.88 ± 7.51	9.08 ± 1.04	3.51
